# Associations of maternal dietary patterns during pregnancy and fetal intrauterine development

**DOI:** 10.3389/fnut.2022.985665

**Published:** 2022-09-15

**Authors:** Rui Qin, Ye Ding, Qun Lu, Yangqian Jiang, Jiangbo Du, Ci Song, Hong Lv, Siyuan Lv, Shiyao Tao, Lei Huang, Xin Xu, Cong Liu, Tao Jiang, Zhixu Wang, Hongxia Ma, Guangfu Jin, Yankai Xia, Zhibin Hu, Feng Zhang, Yuan Lin

**Affiliations:** ^1^State Key Laboratory of Reproductive Medicine, Nanjing Medical University, Nanjing, China; ^2^Department of Epidemiology, Center for Global Health, School of Public Health, Nanjing Medical University, Nanjing, China; ^3^Department of Maternal, Child and Adolescent Health, School of Public Health, Nanjing Medical University, Nanjing, China; ^4^State Key Laboratory of Reproductive Medicine (Suzhou Centre), The Affiliated Suzhou Hospital of Nanjing Medical University, Suzhou Municipal Hospital, Gusu School, Nanjing Medical University, Suzhou, China; ^5^Department of Toxicology and Nutritional Science, School of Public Health, Nanjing Medical University, Nanjing, China; ^6^Department of Biostatistics, School of Public Health, Nanjing Medical University, Nanjing, China; ^7^Key Laboratory of Modern Toxicology of Ministry of Education, School of Public Health, Nanjing Medical University, Nanjing, China; ^8^Obstetrics and Gynecology Hospital, National Health Commission (NHC) Key Laboratory of Reproduction Regulation, Shanghai Institute for Biomedical and Pharmaceutical Technologies, Fudan University, Shanghai, China

**Keywords:** prospective study, pregnancy, dietary patterns, B-ultrasound, intrauterine development

## Abstract

Dietary pattern is excellent in reflecting an individual's eating conditions. Longitudinal data on fetal growth can reflect the process of intrauterine growth. We aimed to evaluate the associations between maternal dietary patterns and intrauterine parameters in middle and late pregnancy. The present study was conducted within Jiangsu Birth Cohort (JBC) study. Dietary information was assessed with a food frequency questionnaire (FFQ) in the second and third trimester of gestation. B-ultrasound scans were performed to obtain fetal intrauterine parameters, including head circumference (HC), femur length (FL), abdominal circumference (AC), and estimated fetal weight (EFW). Exploratory factor analysis was used to extract dietary patterns. Multiple linear regression and linear mixed-effects model (LMM) were used to investigate the association between maternal dietary patterns and fetal growth. A total of 1,936 pregnant women were eligible for the study. We observed inverse associations of maternal “Vegetables and fish” and “Snack and less eggs” patterns during mid-pregnancy with fetal HC Z-score, respectively (“Vegetables and fish”: β = −0.09, 95% CI −0.12, −0.06; “Snack and less eggs”: β = −0.05, 95% CI −0.08, −0.02). On the contrary, “Animal internal organs, thallophyte and shellfish” pattern in the second trimester was associated with increased HC Z-scores (β = 0.04, 95% CI 0.02, 0.06). Consistently, score increase in “Vegetables and fish” pattern in the third trimester was inversely associated with the Z-scores of HC (β = −0.05, 95% CI −0.09, −0.02), while “Meat and less nuts” pattern was positively correlated with the Z-scores of HC (β = 0.04, 95% CI 0.02, 0.07). As compared to the fetus whose mothers at the lowest tertile of “Snack and less eggs” pattern in both trimesters, those whose mothers at the highest tertile demonstrated 1.08 fold (RR = 2.10, 95% CI 1.34–3.28) increased risk of small HC for gestational age (GA). No correlation was observed between maternal dietary patterns and other intrauterine parameters. Our results suggested the effects of maternal dietary patterns on fetal growth, particularly HC. These findings highlighted the adverse impact of unhealthy dietary pattern on fetal growth, might provide evidence for strategies to prevent intrauterine dysplasia and dietary guidelines during pregnancy.

## Introduction

Pregnancy requires an increased intake of energy and macronutrient for maternal and fetal needs (1). Several studies, including observational studies and clinical trials, have shown that maternal energy-protein imbalance, inadequate intake of fatty acid and vitamin B are associated with low birth weight (LBW), preterm birth (PTB), and congenital heart disease in offspring (2–4). Maternal undernutrition and overnutrition have adverse effects on fetal health (5). Even in the absence of malnutrition, maternal diet during pregnancy is paramount in achieving appropriate fetal growth and development (6–8). Previous studies have provided evidence on the associations between individual food or nutrient intake during pregnancy, such as fruits (9) and iron (10), and birth outcomes. However, food and nutrients are not consumed in isolation, and these ingredients in the diet may form complex synergies and interactions (11). The summary description of the overall dietary status better reflects individual's actual eating conditions. Dietary pattern, a semi-quantitative research method describing the overall diet, is particularly suitable for large-scale epidemiological studies (12). Thus, it has gradually become an indispensable method for research on dietary nutrition and health and wellbeing in recent years (13). Several studies have demonstrated the association between maternal dietary patterns and birth out-comes, including the anthropometry measurements of newborns, and the risk of PTB and born small for gestational age (SGA) (14–17).

Compared with the anthropometry measurements of newborns, the longitudinal data of fetal growth measured repeatedly can better reflect the continuous process of intrauterine growth (18). Growth of the fetus *in utero* determines the gratifying outcome of pregnancy, i.e., the birth of a healthy and viable child (19–21). Poor second and third trimester fetal growth has been associated with increased risks of PTB, LBW, and long-term adverse health outcomes (22–24). Normal fetal growth depends on genetic background, endocrine milieu, and the appropriate supply of oxygen and nutrients (25). However, few studies have investigated the influence of maternal dietary patterns during pregnancy on fetus intrauterine development.

Therefore, in the present cohort study of 1,936 mother-infant pairs, we described maternal dietary patterns in mid- to late-pregnancy, and prospectively investigated the associations between maternal dietary patterns and intrauterine growth parameters of fetus.

## Materials and methods

### Study population and design

Our research was based on the Jiangsu Birth Cohort (JBC) study, a prospective and longitudinal study that recruited women who are going to receive assisted reproductive technology (ART) treatment and those who are in their first trimester of spontaneous pregnancy (SP) at the Women's Hospital of Nanjing Medical University or Suzhou Affiliated Hospital of Nanjing Medical University. Detailed cohort design and data collection have been described previously (26). The study was approved by the Human Research Ethics Committee of Nanjing Medical University. The ethical approval code for the project is NJMUIRB (2017) 002. In addition, written informed consent was obtained from all participants.

Since April 2017, the cohort collected maternal dietary information with semi-quantitative food frequency questionnaire (FFQ, including 25-items) in the first [10–14 gestational week (GW)], second (22–26 GW), and third trimester (30–34 GW). From the second trimester of pregnancy to delivery, pregnant women undergo multiple routine ultrasound examinations in the hospital. Based on the distribution of ultrasound examinations time, we chose 3-time points (22–24, 30–32, and 34–36 GW) to achieve as much ultrasound data as possible.

By March 2020, a total of 2,667 single live births were born. Among them, dietary information of 2,183 mothers (81.85%) was collected in the second and third trimester. In addition, 2 mothers with implausible dietary information [total energy intake (TEI) <500 or >5,000 kcal/d] were excluded. Among 2,181 mother-infant pairs with maternal dietary data, 1,936 (88.77%) mothers had at least one B-ultrasound in the designated GWs. The flow chart of participants enrolled in the present study was shown in Supplementary Figure 1.

### Assessment of dietary intakes during pregnancy

Dietary intakes during pregnancy were assessed by semi-quantitative FFQ (Supplementary Table 1), which was evaluated by well-trained investigators with the help of food models and food atlas (27). The responses were reviewed and corrected in time to ensure the completeness and validity of the questionnaires. The daily energy intakes were then calculated according to the Chinese Food Composition Table (28).

Before the analysis, we aggregated the 25 foods into 16 food groups to reduce complexity; these food groups were created based on the expected similar nutrient composition (28). The intake of each food was adjusted for TEI in the second (mean TEI = 2133.1 kcal/d), and third trimester (mean TEI = 2159.0 kcal/d) using the residual method after log-transformation (29). The validity of the FFQ was verified before the formal investigation. One hundred and forty-one pregnant women in middle pregnancy completed both the FFQ and the 3-day 24-h dietary recall (24HR), detailed data have been published elsewhere (30).

### Assessment of outcome

The examination was completed by a professional ultrasound technician performing three ultrasound scans to obtain fetal intrauterine growth data and take the average value. Ultrasound parameters of fetal growth (millimeters) included head circumference (HC), femur length (FL), and abdominal circumference (AC). In addition, gestational age (GA) was calculated according to the interval between the self-reported date of last menstrual period and the date of the B-ultrasound examination. Additionally, estimated fetal weight (EFW), GA adjusted Z-scores and percentiles for fetal growth parameters were calculated according to the International Fetal and Newborn Growth Consortium for the 21st Century (INTER-GROWTH-21st) standards (31, 32). We chose the 10th centile as the cutoff of SGA and the 90th percentile as the large for GA (LGA) cutoff for each parameter since the 10th centile for AC or EFW was used to qualify a fetus as SGA, and the 90th percentile was used to qualify the fetus as LGA in a consensus definition published by an international committee (33).

### Assessment of covariates

Data on mothers (demographic, lifestyle, and clinical factors) and infants (PTB, LBW, sex) were derived from structured questionnaires and electronic medical records (EMR). Questionnaires were collected by face-to-face interviews or telephone. Covariates included mode of conception (SP / ARTP), area of residence (urban, township, rural), household income (<50,000 CNY or 50,000–100,000 CNY or 100,000–200,000 CNY or >200,000 CNY), maternal education (<12/≥12 years), maternal age at conception, maternal pre-pregnancy BMI, parity (primipara / multipara), chronic diabetes (yes/no), gestational diabetes mellitus (GDM, yes / no), TEI, infant sex (male / female). In addition, the dietary patterns are mutually corrected. Notably, only two women reported smoking and eight women reported drinking during pregnancy in this study. Thus, smoking and drinking were not included as covariates.

### Statistical methods

We used exploratory factor analysis to characterize maternal dietary patterns during middle and late pregnancy (34). Dietary patterns were derived by principal component extraction with the use of varimax rotation on the 16 food groups (35). To determine the number of factors to retain, we considered eigenvalues >1 (36), a breakpoint in the Scree test (37) and the interpretability of the factors (38). The dietary patterns identified in the two trimesters were similar in relation to the number of factors identified and the foods that loaded highly (Supplementary Table 2). Therefore, factor analysis was rerun on the geometric mean of food intake during the two pregnancy periods to represent maternal habitual dietary patterns. For each food group, loadings for factors represented the correlation between the food groups and a factor. The dietary patterns were labeled according to food groups that made major contributions to the factor (absolute value of factor loading >0.50 and in the top three of the food groups). Factor scores for each dietary pattern were calculated for each subject with summing the intake of food groups weighted by their factor loadings. In addition, factor loadings are correlation coefficients between each food group and the dietary pattern; hence, higher dietary pattern scores indicate greater adherence to the derived pattern (39).

Baseline characteristics were described as percentages or mean (SD). According to the tertiles of dietary pattern scores, all pregnant women were divided into three groups to compare the distribution of macronutrient intake. We performed linear trends across tertiles using linear regression (median intake for each tertile as variables included in the model). The intraclass correlation coefficient (ICC) and 95% CI were calculated by dietary pattern scores to assess the temporal variability of dietary patterns during pregnancy. The linear mixed-effects model (LMM) was used to examine the associations between maternal dietary pattern scores in the two trimesters and longitudinal indicators of intrauterine development (40). Analyses were adjusted for mode of conception (SP / ARTP), area of residence (urban, township, rural), household income (<50,000 CNY or 50,000–100,000 CNY or 100,000–200,000 CNY or >200,000 CNY), maternal education (<12/≥12 years), maternal age at conception (year), maternal pre-pregnancy BMI (continuous), parity (primipara / multipara), chronic diabetes (yes/no), GDM (yes/no), TEI, infant sex (male / female). In addition, the dietary patterns were mutually corrected. False discovery rate (FDR) (41) was utilized to correct for multiple tests, and FDR-*p* < 0.05 was set as the significance threshold. To avoid the potential confounding effects of maternal diabetes and anemia, we excluded mothers with such conditions in the sensitivity analyses. Maternal diabetes included preexistent diabetes and GDM (42), and anemia was defined as hemoglobin levels <110 g/L (43). In addition, a stratified analysis was performed according to the mode of conception, and heterogeneity was tested. All analyses were conducted in R software (Version 3.6.1, R Foundation for Statistic Computing, Vienna, Austria. URL https://www.R-project.org/).

## Results

### Basic characteristics

Demographic characteristics of the 1,936 enrolled mother-infant pairs were summarized in Table 1. The JBC study was originally designed to investigate the heterogeneity of assisted vs. natural pregnancy in perinatal outcomes and child health, with 45.8% (*n* = 887) of mothers conceived after assisted reproduction in this analysis. The majority of women live in cities (79.3%) and a medium socioeconomic status level household (63.8%). Fewer than one in five women have <12 years of education (18.4%). Approximately two-thirds (*n* = 1,311) of women within a normal weight before pregnancy and 246 (12.7%) women were over 35 years old at conception. In addition, 1,531 (79.1%) mothers were primiparous. And the number of mother with GDM and anemia were 517 (26.7%) and 188 (9.71%), respectively. Baseline information on offspring indicated that the incidence of PTB (GA <37 GW) and LBW (birth weight <2,500 g) was 4.2% (*n* = 81) and 2.7% (*n* = 53), respectively. The proportion of male infants (52.4%) was slightly higher than that of female infants.

**Table 1:** Characteristics of baseline demographic and lifestyle factors of 1936 mother-infant pairs.

**Mothers' characteristics**	**Overall**	**Offspring's characteristics**	**Overall**
**Mode of conception**		GW at delivery	39.43 (1.35)
SP	1,049 (54.2)	PTB*	81 (4.2)
ARTP	887 (45.8)	Birth weight (g)	3384.35 (443.31)
**Area of residence**		LBW (<2,500 g)	53 (2.7)
Urban	1,530 (79.3)		
Township	290 (15.0)	Male birth	1,014 (52.4)
Rural	110 (5.7)		
**Household income (CNY)**		HC (cm)	
<50,000	69 (3.6)	22–24 GW	21.39 (0.98)
50,000–100,000	435 (22.6)	30–32 GW	28.90 (1.15)
100,000–200,000	794 (41.2)	34–36 GW	31.51 (1.14)
>200,000	629 (32.6)		
Maternal education (years) <12	355 (18.4)	AC (cm)	
**Maternal pre-pregnancy BMI (kg/m** ^**2**^ **)**		22–24 GW	19.26 (1.17)
<18.5	230 (12.0)	30–32 GW	27.69 (1.36)
18.5–23.9	1,311 (68.3)	34–36 GW	31.60 (1.51)
24–27.9	306 (15.9)		
≥28	72 (3.8)	FL (cm)	
Maternal age at conception (years) ≥ 35	246 (12.7)	22–24 GW	4.16 (0.25)
Primipara	1,531 (79.1)	30–32 GW	5.97 (0.26)
Chronic diabetes	14 (0.7)	34–36 GW	6.72 (0.27)
GDM	517 (26.7)		
Anemia	188 (9.71)	EFW (g)	
**Maternal TEI (100 kcal/d)**		22–24 GW	668.58 (77.21)
Second trimester	21.33 (5.33)	30–32 GW	1742.28 (240.95)
Third trimester	21.59 (5.36)	34–36 GW	2574.98 (335.38)

*Gestational age (GA) <37 GW.

SP, spontaneous pregnancy; ARTP, assisted reproductive technology pregnancy; GDM, gestational diabetes mellitus; TEI, total energy intake; GW, gestational week; PTB, preterm birth; LBW, low birth weight; HC, head circumference; AC, abdominal circumference; FL, femur length; EFW, estimated fetal weight.

### Maternal dietary patterns

**Figure 1:**
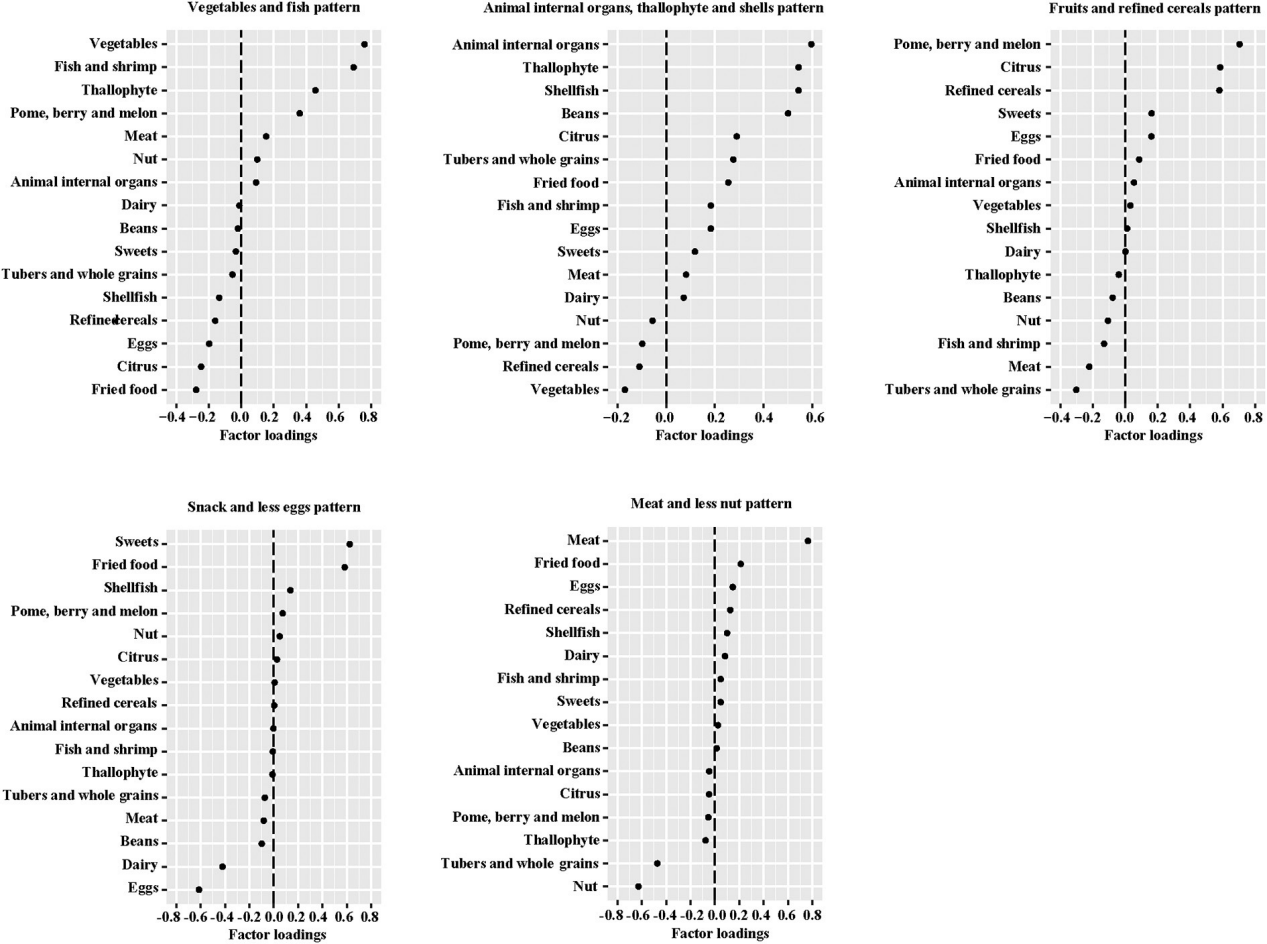
Factor loadings of averaged* food groups in middle and late pregnancy for five dietary patterns. *The geometric mean of food groups in two trimesters of pregnancy.

Five dietary patterns were identified by using factor analysis (Figure 1), which accounted for 46.4% of the total changes in dietary intake. The “Vegetables and fish” pattern was characterized by higher intakes of dietary fiber, minerals, and high-quality protein, explaining 10.2% of the variation in the dietary data. The “Animal internal organs, thallophyte and shellfish” pattern, which explained 10.0% of the variation, is rich in micronutrients, cholesterol and animal protein. In addition, “Fruits and refined grains” pattern, “Snack and less eggs” pattern, and “Meat and less nuts” pattern explained 9.1, 8.1, and 8.0% of the variation, respectively. In the evaluation of temporal variability of diets across pregnancy, all dietary patterns showed high consistency (ICC > 0.40) from mid- to late-gestation in our study population (Supplementary Table 3), especially the “Vegetables and fish” pattern (ICC = 0.631). Macronutrient intakes of 1,936 mothers were described according to tertiles of dietary pattern scores (Supplementary Table 4). Compared to women in the lowest tertile of the “Vegetables and fish” pattern, women in the highest tertile had higher intakes of protein (second trimester: 18.77% compared with 16.15% energy; third trimester: 18.90% compared with 16.57% energy) and dietary fiber (second trimester: 7.29 g compared with 6.51 g/1,000 kcal; third trimester: 7.34 g compared with 6.25 g/1,000 kcal). We observed that women with a greater adherence to the “Animal internal organs, thallophytic and shellfish” pattern or “Meat and less nuts” pattern (third tertile) had a higher mean intake of cholesterol. Women in the highest tertile of “Snack and less eggs” pattern score had the lowest cholesterol intake than all other tertiles of dietary patterns. In addition, protein intake declined along with the increased score of “Snack and less eggs” pattern (*p* for trend < 0.001).

### Maternal dietary patterns and fetal growth indicators

Associations of maternal dietary patterns in the second and third trimester with fetal growth index were shown in Table 2. In the second trimester, maternal “Vegetables and fish” (β = −0.09, 95% CI −0.12, −0.06) and “Snack and less eggs” (β = −0.05, 95% CI −0.08, −0.02) pattern were associated with decreased fetal HC Z-score from mid-to late-gestation. On the contrary, each score increases in “Animal internal organs, thallophyte and shellfish” pattern was associated with 0.04 (0.02, 0.06) in Z-scores of fetal HC. We then investigated maternal dietary patterns in the third trimester in relation to intrauterine growth parameters in late pregnancy. Consistently, per score increase in “Vegetables and fish” pattern in the third trimester was inversely associated with the Z-scores of HC (β = −0.05, 95% CI −0.09, −0.02) in late pregnancy, while “Meat and less nuts” pattern was positively correlated with the Z-scores of HC (β = 0.04, 95% CI 0.02, 0.07) were positively correlated with this pattern score. However, no correlation was observed between maternal dietary patterns and AC, FL, and EFW. In addition, we investigated maternal dietary patterns in relation to offspring birth weight after further adjusting for GW at delivery (Supplementary Table 5). No significant association was observed between maternal dietary patterns during pregnancy and offspring birth weight. We further carried out sensitivity analyses by excluding women who were complicated with chronic diabetes or GDM (Supplementary Table 6), as well as women who were diagnosed with anemia during pregnancy (Supplementary Table 7), and the main results remained stable. In addition, we conducted stratified analyses by mode of conception (Supplementary Table 8). Though some associations were not statistically significant when splitting the study population, the main results were consistent in both groups and no heterogeneity was observed.

**Table 2:** Adjusted associations of maternal dietary patterns in the second and third trimester of pregnancy with fetal growth indicators.

**Dietary patterns**	**HC Z-score**		**AC Z-score**		**FL Z-score**		**EFW Z-score**	
	**Beta (95% CI)**	**FDR-*p***	**Beta (95% CI)**	**FDR-*p***	**Beta (95% CI)**	**FDR-*p***	**Beta (95% CI)**	**FDR-*p***
Second trimester
Vegetables and fish	**−0.09 (−0.12, −0.06)**	**<0.001**	0.004 (−0.02, 0.03)	0.959	−0.001 (−0.03, 0.03)	0.998	−0.02 (−0.05, 0.01)	0.332
Animal internal organs, thallophyte and shellfish	**0.04 (0.02, 0.06)**	**0.002**	0.01 (−0.01, 0.03)	0.523	−0.001 (−0.02, 0.02)	0.998	0.02 (−0.001, 0.04)	0.181
Fruits and refined grains	−0.01 (−0.04, 0.02)	0.748	0.01 (−0.01, 0.04)	0.613	−0.01 (−0.03, 0.02)	0.898	0.01 (−0.02, 0.03)	0.851
Snack and less eggs	**−0.05 (−0.08, −0.02)**	**0.002**	−0.01 (−0.03, 0.02)	0.748	−0.01 (−0.04, 0.02)	0.650	−0.02 (−0.05, 0.01)	0.291
Meat and less nuts	0.03 (0.01, 0.06)	0.146	−0.01 (−0.04, 0.01)	0.542	0.01 (−0.01, 0.04)	0.638	−0.005 (−0.03, 0.02)	0.922
Third trimester
Vegetables and fish	**−0.05 (−0.09, −0.02)**	**0.009**	0.01 (−0.02, 0.04)	0.803	0.01 (−0.03, 0.04)	0.896	−0.004 (−0.03, 0.02)	0.896
Animal internal organs, thallophyte and shellfish	0.01 (−0.01, 0.04)	0.615	−0.01 (−0.03, 0.01)	0.700	−0.01 (−0.03, 0.01)	0.700	−0.005 (−0.03, 0.02)	0.876
Fruits and refined grains	−0.03 (−0.06, 0.01)	0.219	0.002 (−0.02, 0.03)	0.963	0.001 (−0.03, 0.03)	0.963	−0.005 (−0.03, 0.02)	0.896
Snack and less eggs	−0.04 (−0.07, −0.01)	0.133	0.005 (−0.02, 0.03)	0.896	−0.03 (−0.06, 0.01)	0.349	−0.005 (−0.03, 0.02)	0.896
Meat and less nuts	**0.04 (0.02, 0.07)**	**0.013**	−0.001 (−0.03, 0.02)	0.963	0.03 (0.01, 0.05)	0.165	0.01 (−0.01, 0.03)	0.751

Analyses were adjusted for mode of conception, area of residence, household income, maternal education, maternal age at conception, maternal pre-pregnancy BMI, parity, chronic diabetes, GDM, TEI, and infant sex. In addition, the dietary patterns were adjusted for each other.

HC, head circumference; AC, abdominal circumference; FL, femur length; EFW, estimated fetal weight; FDR, false discovery rate; TEI, total energy intake; GDM, gestational diabetes mellitus.

Bold values means a statistically significant difference in results.

As these dietary patterns were significantly associated with fetal HC following adjustment for covariates, Supplementary Table 9 illustrates the associations of fetal HC with intakes of food groups. After adjusting for covariates, the effects of food intake on fetal HC remained largely consistent with the dietary patterns it constituted, although not all statistically significant.

### Adherence to the same dietary pattern in both trimesters and fetal HC

To assess the effect of adherence to the same dietary pattern from middle to late pregnancy and fetal HC, we categorized participants into tertiles according to their scores on the dietary patterns in both second and third trimester (Table 3). As compared to the fetus whose mothers at the lowest tertile of the score on “Vegetables and fish” pattern in both trimesters, those whose mothers at the highest tertile demonstrated 0.28 decreased Z-score in fetal HC (β = −0.28, 95% CI −0.43, −0.14). Similar associations exist between “Snack and less eggs” pattern and HC Z-score. Additionally, HC Z-score of the fetus in the highest tertile of the mother's “Meat and less nuts” pattern increased by 0.18 (0.04, 0.33) as compared with the lowest tertile.

**Table 3:** Adjusted associations of tertiles of dietary patterns in the second and third trimester with fetal HC Z-score.

**Dietary patterns**	***N***	**HC Z-score**	
		**Beta (95% CI)**	**FDR*-p***
Vegetables and fish
Lowest tertile in both trimesters	343	Ref	
Highest tertile in both trimesters	368	**−0.28 (−0.43, −0.14)**	**0.001**
Animal internal organs, thallophyte and shellfish
Lowest tertile in both trimesters	322	Ref	
Highest tertile in both trimesters	349	0.08 (−0.06, 0.22)	0.326
Fruits and refined grains
Lowest tertile in both trimesters	304	Ref	
Highest tertile in both trimesters	311	−0.01 (−0.16, 0.15)	0.926
Snack and less eggs
Lowest tertile in both trimesters	335	Ref	
Highest tertile in both trimesters	350	**−0.19 (−0.33, −0.05)**	**0.016**
Meat and less nuts
Lowest tertile in both trimesters	299	Ref	
Highest tertile in both trimesters	316	**0.18 (0.04, 0.33)**	**0.019**

Analyses were adjusted for mode of conception, area of residence, household income, maternal education, maternal age at conception, maternal pre-pregnancy BMI, parity, chronic diabetes, GDM, TEI, and infant sex. In addition, the dietary patterns were adjusted for each other. Ref, reference group, which is the group of mothers with the lowest tertile of dietary pattern scores in both trimesters.

HC, head circumference; FDR, false discovery rate; TEI, total energy intake; GDM, gestational diabetes mellitus.

Bold values means a statistically significant difference in results.

We observed 10.66% of HC in late pregnancy below the 10th centile and 7.59% above the 90th centile with INTERGROWTH-21st. Further analyses investigating associations of dietary patterns with small and large HC for GA were carried out. As compared to the fetus whose mothers at the lowest tertile of “Snack and less eggs” pattern in both trimesters, those whose mothers at the highest tertile in both trimesters demonstrated 1.08 fold (RR = 2.10, 95% CI 1.34–3.28) increased risk of small HC for GA after adjusting for potential confounders (Table 4). No significant associations were observed between dietary patterns and the risk of large HC for GA.

**Table 4:** Adjusted associations of tertiles of dietary patterns in the second and third trimester with small and large HC for GA of fetus.

**Dietary patterns**	***N***	**Small HC for GA**		**Large HC for GA**	
		**RR (95% CI)**	**FDR*-p***	**RR (95% CI)**	**FDR*-p***
**Vegetables and fish**
Lowest tertile in both trimesters	343	Ref		Ref	
Highest tertile in both trimesters	368	1.40 (0.93, 2.10)	0.556	0.44 (0.25, 0.80)	0.061
**Animal internal organs, thallophyte and shellfish**
Lowest tertile in both trimesters	322	Ref		Ref	
Highest tertile in both trimesters	349	0.84 (0.56, 1.26)	0.900	0.86 (0.52, 1.43)	0.939
**Fruits and refined grains**
Lowest tertile in both trimesters	304	Ref		Ref	
Highest tertile in both trimesters	311	0.83 (0.52, 1.33)	0.900	0.78 (0.44, 1.38)	0.900
**Snack and less eggs**
Lowest tertile in both trimesters	335	Ref		Ref	
Highest tertile in both trimesters	350	**2.10 (1.34, 3.28)**	**0.013**	1.03 (0.63, 1.69)	0.979
**Meat and less nuts**
Lowest tertile in both trimesters	299	Ref		Ref	
Highest tertile in both trimesters	316	0.60 (0.39, 0.93)	0.182	1.27 (0.71, 2.25)	0.900

Analyses were adjusted for mode of conception, area of residence, household income, maternal education, maternal age at conception, maternal pre-pregnancy BMI, parity, chronic diabetes, GDM, TEI, and infant sex. In addition, the dietary patterns were adjusted for each other. Ref, reference group, which is the group of mothers with the lowest tertile of dietary pattern scores in both trimesters.

HC, head circumference; GA, gestational age; FDR, false discovery rate; TEI, total energy intake; GDM, gestational diabetes mellitus.

Bold values means a statistically significant difference in results.

## Discussion

The present study prospectively investigated the associations between maternal dietary patterns across the second to third trimester and intrauterine growth parameters in middle and late pregnancy in a Chinese birth cohort study. Notably, our study demonstrated the positive associations of maternal “Animal internal organs, thallophyte and shellfish” and “Meat and less nuts” patterns with fetal HC; and the negative correlation between “Vegetables and fish” and “Snack and less eggs” pat-terns and fetal HC. Further, these effects persisted after we excluded mothers with diabetes or anemia. No heterogeneity was observed in this association between groups of different conception methods, which suggested the effects of dietary patterns on fetal growth were not significantly different among ART and SP populations. We cannot exclude the possibility of the relatively modest sample size in the stratification analyses causing underestimation of the significance of true associations due to statistical power (44). In addition, the effects were more significant if one adhered to the same pattern across the second and third trimester. It should also be noted that high adherence to the “Snack and less eggs” pattern increases the risk of small HC for GA. However, no correlation was observed between maternal dietary patterns and AC, FL, EFW and birth weight.

“Animal internal organs, thallophyte and shellfish” and “Meat and less nuts” patterns are rich in meat and meat products, which contribute significantly to the intake of cholesterol, protein and essential minerals such as iron and zinc (45). Cholesterol plays a pivotal role in many aspects of brain development. The brain has the highest cholesterol content compared with other organs (25% of total body cholesterol), while representing only about 5% of total body weight (46). Analysis of 5,702 pregnant women in the Generation R Study showed that lipid levels in the first trimester were positively associated with neonatal HC (47). Additionally, zinc has multiple roles in brain growth, differentiation, and repair (48). One prospective study of 7,644 pregnant women in Foshan, China also showed that maternal serum zinc levels at 24 GW were positively correlated with neonatal HC (49). Notably, the level of zinc in shellfish is much higher than that in fish and shrimp, which may lead to their different effects on HC (28). Observational study including 538 children aged 3–4 in rural Nepal showed a positive correlation between animal food intake and HC (50). Clinical intervention trial of 88 infants further demonstrated that the increase in HC from 7 to 12 months for supplemented with meat group was higher than cereal-complementary group, and protein and zinc intakes were predictors of head growth (51). One observational study assessed offspring's HC among vegetarian and omnivorous pregnant women and reported that offspring's HC of vegetarian mothers was smaller than that of omnivorous mothers (52), which also suggested the potential effects of animal protein on fetal HC. Thallophyte and shellfish are not only rich in protein and micronutrients, but also a quality source of iodine (53, 54). Iodine is an essential micronutrient and a component of the thyroid hormones, which regulate growth and development from conception to adulthood (55, 56). A study conducted in a pregnancy cohort of 2087 women found that high urinary iodine concentration in the second trimester was associated with higher HC of fetal during pregnancy (57). Our findings are in line with the above studies, and the adherence to “Meat and less nuts” pattern across both trimesters demonstrated more distinct effects on increased fetal HC.

Moreover, we found that maternal “Vegetables and fish” and “Snack and less eggs” patterns had a lowering effect on the HC of fetus, the latter also increasing the risk of small HC for GA. Fiber-rich in “Vegetables and fish” pattern increases post-meal satiety and reduces food intake behavior (58). Fish and shrimp were not only rich in nutrients such as marine n-3 fatty acids, vitamin D, and selenium, but can also be a source of pollutants such as methylmercury, arsenic and polychlorinated biphenyls (59, 60). Study based on data from the Child Health and Development Study in the San Francisco Bay Area found that high polychlorinated biphenyl exposure *in utero* was associated with reduced HC at birth (61). As reported by the China Fisheries Statistical Yearbook (62), lean fish species are common food fish in Jiangsu Province, China (62). One prospective cohort study in Italy of 114 mother-infant pairs reported a negative association between maternal consumption of lean fish during pregnancy and neonatal HC (63). Notably, thallophyte and shellfish may contain mercury and persistent pollutants, but meanwhile their nutrient substances such as high levels of zinc and iodine are beneficial for fetal growth (28, 53, 54). The “Snack and less eggs” pattern is an unhealthy diet characterized by low cholesterol and protein. Results from a cohort study of 1,151 women in the southern US demonstrated that adherence to a dietary pattern characterized by fast food, snacks, sweets, and soft drinks during pregnancy reduced offspring head neonatal HC (64). In addition, snack food products are often nutrient-poor, high in salt or sugar, and not recommended for pregnant women (65). Similar to cholesterol (46), protein plays an indispensable role in the development of fetal brain anatomy and physiology (66). A randomized controlled parenteral nutrition study in very early preterm infants also showed that post-natal high-protein diet could improve offspring's HC at 28 days (67).

It was worth noting that our results suggested effects of maternal dietary patterns with growth index in fetal being observed only associated with HC, but not with AC, FL, EFW, and birth weight. Head growth is an independent process and proceeds independently of skeletal growth and fat acquisition (68). FL, AC and body weight are not effective indicators for evaluating head development. Randomized controlled trial of 196 women showed that dietary and lifestyle interventions during pregnancy were associated with offspring's HC but not offspring's weight, and the association persisted until offspring 1-year-old (69). A pilot trial of portable ultrasound with 47 second-trimester women in Ecuador found a significant association between maternal diet and fetal HC, while EFW was not affected by maternal diet (70). In addition, two birth cohort studies in the U.S. reported that dietary pattern in the mid- to late-trimester has no effect on the birth length or weight of offspring (64, 71), but were associated with HC at birth (64). As far as AC was concerned, the dietary pattern in the third trimester of pregnancy has no effect on the AC of the offspring even up to 54 months of age (72).

A key strength of our study is the longitudinal evaluation of embryonic growth, providing data on fetal growth across pregnancy. Further, prospective and longitudinal data collection allowed examining the temporal associations of maternal dietary patterns and subsequent fetal development. In addition, the use of dietary patterns, which provides an insight into the overall quality of the diet, a feature single-food or nutrient studies cannot provide (12). Finally, a wide range of potential confounding factors was evaluated and controlled in the analysis. Some potential limitations of the research merit discussion. First, the limited items in the FFQ and is prone to be biased by poor participant recall of dietary intake. The FFQ used in this study was scientifically designed and its implementation process was strictly controlled. Moreover, we have confirmed the high validity of the questionnaire in a pre-survey. Second, we did not include maternal B-ultrasound data in the first tri-mester in the current study as the B-ultrasound before 14 GW only provides the crown-rump length (CRL) to assess the GA and fetal size. Additionally, meta-analysis also showed that ultrasound data in the middle and late trimesters rather than the early trimester have predictive value for birth outcomes (73). Third, we did not collect HC, AC and FL at birth, which hindered the evaluation of accuracy of intrauterine ultrasound parameters. However, the EFW and birth weight showed well consistency (data not shown), which may reflect the accuracy of B-ultrasound data to some extent. Finally, despite the strengths of our study, its findings should be interpreted with some caution. Our survey was conducted in China, where food culture is significantly different from other countries, especially Western countries. Therefore, the generalization of our findings to other countries remains to be established.

## Conclusion

In the prospective and longitudinal JBC study, our results suggested the effects of maternal dietary patterns on fetal growth, particularly HC. These findings highlighted the adverse impact of unhealthy dietary pattern during pregnancy on fetal growth, might provide evidence for strategies to prevent intrauterine dysplasia and dietary guidelines during pregnancy. This study also validates the practicality of “dietary pattern” for research and health guidance, which takes into account the correlation structure of the food groups and does not focus on selected aspects of a diet. The observed associations between maternal dietary patterns and fetal growth highlighted research on maternal nutrition evaluated by “dietary pattern” in the field of intrauterine growth and even post-natal health. Furthermore, our findings may have important clinical and public health implications. Considering the high prevalence of fetal growth restriction and its potential negative impact on lifelong health, improving maternal diet is of utmost importance. Therefore, awareness of the importance of a healthy dietary pattern should be raised among pregnant women. Future prospective studies with longer follow-up are warranted to determine whether maternal dietary patterns may impact longer-term child growth beyond intrauterine development.

## Data availability statement

The raw data supporting the conclusions of this article will be made available by the authors, without undue reservation.

## Ethics statement

The studies involving human participants were reviewed and approved by the Human Research Ethics Committee of Nanjing Medical University. The patients/participants provided their written informed consent to participate in this study.

## Author contributions

YL, RQ, YD, and QL: conceptualization. RQ: methodology and writing original draft preparation. RQ and YL: formal analysis. RQ, QL, YD, YJ, JD, CS, HL, SL, ST, LH, XX, CL, and ZW: investigation. ZH and YL: resources and funding acquisition. TJ, HM, GJ, YX, YL, FZ, and ZH: data curation. All authors contributed to the article and approved the submitted version.

## Funding

This manuscript and research were supported by the National Key Research & Development (R&D) Program of China (2021YFC2700705) and the National Nature Science Foundation of China (82103919).

## Conflict of interest

The authors declare that the research was conducted in the absence of any commercial or financial relationships that could be construed as a potential conflict of interest.

## Publisher's note

All claims expressed in this article are solely those of the authors and do not necessarily represent those of their affiliated organizations, or those of the publisher, the editors and the reviewers. Any product that may be evaluated in this article, or claim that may be made by its manufacturer, is not guaranteed or endorsed by the publisher.
