# Supplementary material for: Associations of maternal dietary patterns during pregnancy and fetal intrauterine development
Source: Front Nutr. 2022 Sep 15;9:985665. doi: 10.3389/fnut.2022.985665 (PMC9520705; doi:10.3389/fnut.2022.985665)
Supplement: Supplementary file 1 [file Data_Sheet_1.docx]

Supplementary Material

****Supplemental file 1: Supplementary Figure 1****

****Supplemental file 2: Supplementary Table 1-9****


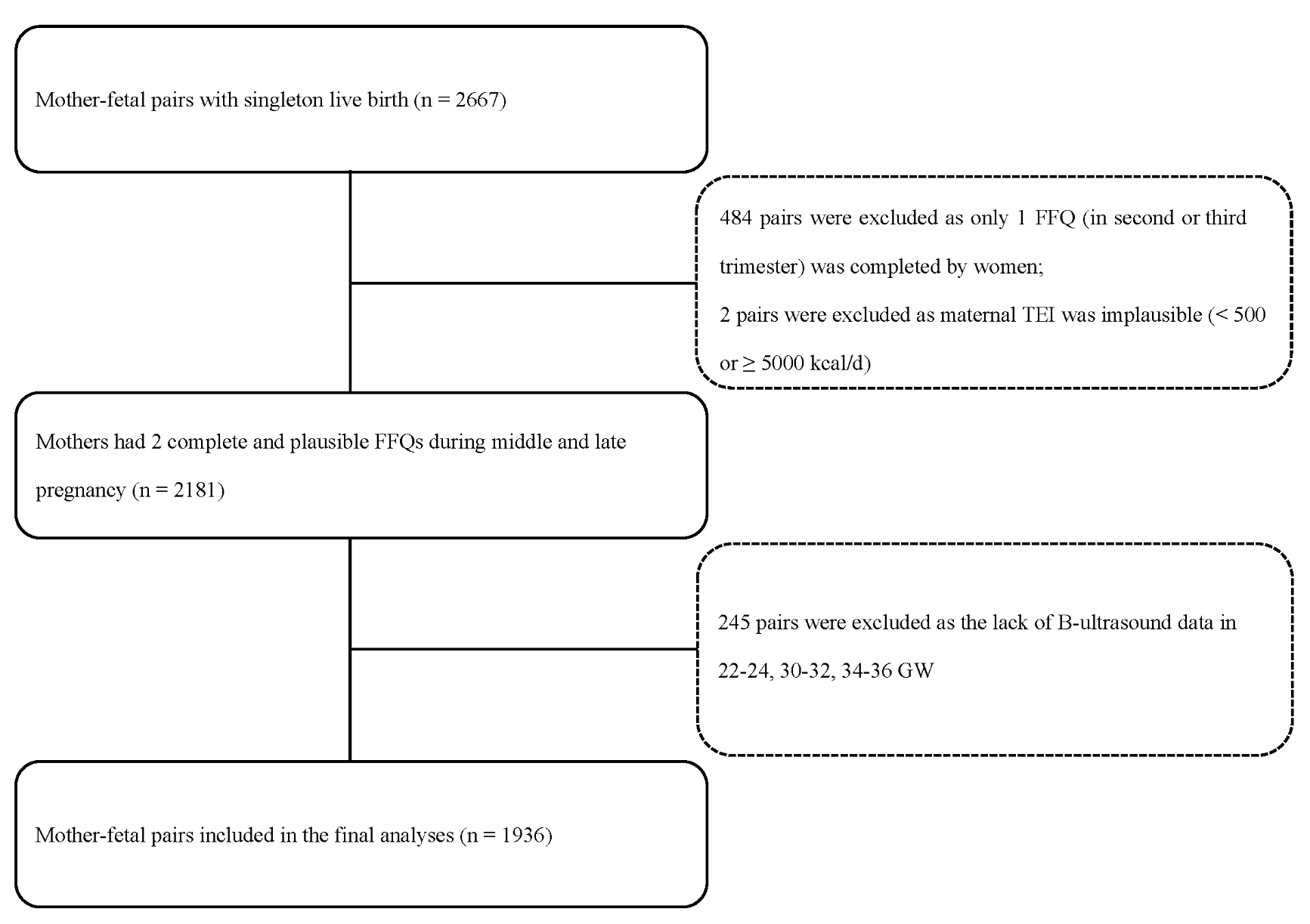


**Supplementary Figure 1. Flow chart of the prospective cohort study.**

Abbreviations: FFQ, food frequency questionnaire; GW, gestational week.

| **Supplementary Table 1. Food group list in the FFQ.** | |
| --- | --- |
| **Order** | **Food groups** |
| 1 | Rice, porridge, rice noodles and their products |
| 2 | Wheat products such as noodles, steamed buns, flower rolls and sesame seeds (excluding fried noodles) |
| 3 | Chinese fried dough foods (deep-fried dough sticks etc.) |
| 4 | Tubers such as potatoes, sweet potatoes, taro, yams, vermicelli and their products |
| 5 | Millet, corn, beans, sorghum, oats and other grains |
| 6 | Tofu, thousand sheets, dried tofu, tofu brain, soy milk and other soy products |
| 7 | Dried beans and soy flour |
| 8 | Bacteria and algae such as mushrooms, enoki mushrooms, fungus, kelp and seaweed |
| 9 | Root, stem, leaf, flower and sprout vegetables |
| 10 | Citrus fruits such as tangerines, oranges and lemons |
| 11 | Fruits such as berries, pome fruits, melons and tropical fruits |
| 12 | Red dates, longan, dried bananas and other dried fruits |
| 13 | Nuts such as peanuts, walnuts, cashews and melon seeds |
| 14 | Liver, kidney, heart and other animal organs |
| 15 | Pig blood, sheep blood, duck blood and other animal blood products |
| 16 | Livestock and poultry meat such as pork, beef, lamb, chicken and duck (excluding animal organs and blood products) |
| 17 | Shrimp and Crabs |
| 18 | Fish such as marine and freshwater fish |
| 19 | Shellfish such as abalone, scallops, clams and screws |
| 20 | Mollusks such as jellyfish and squid |
| 21 | Eggs such as chicken, duck and goose eggs |
| 22 | Dairy such as fresh milk, yogurt and powdered milk |
| 23 | Bread, biscuits, cakes, moon cakes and other pastries |
| 24 | Sweets such as candy and chocolate |
| 25 | Puffed food such as potato chips and hollow chips |
| FFQ, food frequency questionnaire. | |

| **Supplementary Table 2. Factor loadings of food groups in middle and late pregnancy.** | | | | | | | | | | | | | |
| --- | --- | --- | --- | --- | --- | --- | --- | --- | --- | --- | --- | --- | --- |
| **Food groups** | **Second trimester** | | | | | |  | **Third trimester** | | | | | |
|  | **Vegetables, fish and thallophyte** | **Animal internal organs and shellfish** | **Fruits** | **Snack** | **Nut, whole grain and less meat** | **Dairy and eggs** |  | **Vegetables and fish** | **Pome, refined cereals and less whole grain** | **Snack and shellfish** | **Animal internal organs** | **Whole grain and less meat** | **Eggs and dairy** |
| **Refined cereals** | -0.154 | -0.125 | 0.496 | 0.109 | -0.144 | -0.011 |  | -0.133 | **0.604** | 0.121 | -0.094 | 0.007 | 0.143 |
| **Tubers and whole grains** | 0.098 | 0.245 | -0.065 | -0.061 | **0.513** | -0.120 |  | 0.074 | **-0.545** | 0.125 | -0.104 | **0.522** | 0.186 |
| **Fried food** | -0.192 | 0.222 | 0.080 | **0.645** | -0.124 | -0.074 |  | -0.144 | 0.080 | **0.660** | 0.018 | -0.020 | -0.186 |
| **Sweets** | 0.075 | 0.002 | 0.048 | **0.769** | 0.119 | 0.034 |  | 0.083 | 0.237 | **0.557** | -0.010 | 0.101 | -0.045 |
| **Beans** | 0.138 | 0.445 | 0.003 | 0.210 | 0.061 | 0.137 |  | 0.068 | -0.246 | 0.137 | 0.347 | -0.043 | 0.343 |
| **Vegetables** | **0.693** | -0.197 | 0.046 | -0.097 | -0.101 | -0.075 |  | **0.743** | 0.178 | -0.178 | -0.178 | 0.005 | -0.054 |
| **Thallophyte** | **0.546** | 0.375 | -0.066 | 0.126 | 0.035 | 0.011 |  | 0.483 | -0.130 | 0.209 | 0.407 | 0.129 | 0.231 |
| **Citrus** | -0.232 | 0.268 | **0.573** | -0.059 | 0.145 | 0.013 |  | -0.341 | 0.419 | 0.155 | 0.455 | 0.086 | 0.002 |
| **Pome, berry and melon** | 0.383 | -0.183 | **0.603** | 0.052 | 0.135 | -0.144 |  | 0.191 | **0.698** | 0.005 | -0.001 | 0.094 | -0.038 |
| **Nut** | -0.104 | -0.025 | -0.461 | -0.052 | **0.515** | -0.091 |  | 0.004 | 0.018 | -0.363 | 0.469 | 0.395 | -0.390 |
| **Eggs** | -0.041 | 0.206 | 0.219 | -0.323 | 0.015 | **0.650** |  | -0.185 | 0.030 | -0.147 | 0.028 | 0.037 | **0.670** |
| **Dairy** | 0.055 | -0.125 | -0.209 | 0.165 | -0.045 | **0.752** |  | 0.112 | 0.051 | -0.082 | -0.019 | -0.024 | **0.593** |
| **Meat** | 0.092 | 0.198 | -0.199 | -0.150 | **-0.768** | -0.162 |  | 0.031 | -0.128 | 0.011 | 0.049 | **-0.874** | 0.048 |
| **Animal internal organs** | 0.028 | **0.620** | -0.091 | 0.010 | -0.067 | -0.013 |  | 0.077 | -0.013 | 0.047 | **0.711** | -0.119 | -0.010 |
| **Fish and shrimp** | **0.670** | 0.110 | -0.126 | -0.051 | 0.029 | 0.098 |  | **0.705** | -0.091 | 0.001 | 0.166 | -0.039 | -0.024 |
| **Shellfish** | -0.071 | **0.601** | 0.052 | 0.005 | 0.048 | -0.051 |  | -0.034 | -0.138 | **0.531** | 0.115 | -0.051 | 0.013 |

| **Supplementary Table 3. Distribution and ICC of dietary pattern scores across gestation.** | | |  |
| --- | --- | --- | --- |
| **Dietary patterns** | **Median (IQR)** | | **ICC (95% CI)** |
|  | **Second trimester** | **Third trimester** |  |
| **Vegetables and fish** | 7.57 (6.55, 8.44) | 7.77 (6.84, 8.60) | 0.631 ( 0.602, 0.658 ) |
| **Animal internal organs, thallophyte and shellfish** | 6.64 (5.41, 7.85) | 6.87 (5.63, 8.01) | 0.623 ( 0.594, 0.650 ) |
| **Fruits and refined grains** | 6.76 (5.79, 7.49) | 6.53 (5.21, 7.30) | 0.475 ( 0.434, 0.513 ) |
| **Snack and less eggs** | -3.19 (-3.75, -2.22) | -3.29 (-3.87, -2.38) | 0.622 ( 0.593, 0.649 ) |
| **Meat and less nuts** | 1.88 (1.15, 2.81) | 1.99 (1.14, 3.09) | 0.508 ( 0.470, 0.543 ) |
| Abbreviations: IQR, interquartile range; ICC, intraclass correlation coefficient. | | |  |

| **Supplementary Table 4. Macronutrient intakes of 1936 mothers according to tertiles of dietary pattern scores.** | | | | | | | | | | | | | | | |
| --- | --- | --- | --- | --- | --- | --- | --- | --- | --- | --- | --- | --- | --- | --- | --- |
| **Macronutrient intakes** | **Vegetables and fish** | | | **Animal internal organs, thallophyte and shellfish** | | | **Fruits and refined grains** | | | **Snack and less eggs** | | | **Meat and less nuts** | | |
|  | **T1** | **T3** | ***p*-trend*** | **T1** | **T3** | ***p*-trend** | **T1** | **T3** | ***p*-trend** | **T1** | **T3** | ***p*-trend** | **T1** | **T3** | ***p*-trend** |
| **Second trimester** |  |  |  |  |  |  |  |  |  |  |  |  |  |  |  |
| **TEI (100kcal/d)** | **21.29 (5.91)** | **21.92 (4.85)** | **0.046** | 21.43 (5.85) | 21.37 (4.95) | 0.823 | 21.07 (5.49) | 21.18 (5.32) | 0.517 | 20.51 (4.59) | 20.85 (5.93) | 0.651 | 20.88 (5.34) | 20.51 (4.78) | 0.080 |
| **Protein, % energy** | **16.15 (2.77)** | **18.77 (2.59)** | **<0.001** | **17.14 (3.17)** | **18.08 (2.63)** | **<0.001** | **18.31 (2.77)** | **16.44 (2.75)** | **<0.001** | **18.35 (2.58)** | **16.49 (3.14)** | **<0.001** | **16.10 (2.89)** | **18.67 (2.53)** | **<0.001** |
| **Total fat, % energy** | **40.42 (5.50)** | **42.01 (4.54)** | **<0.001** | 41.63 (5.60) | 41.17 (4.51) | 0.093 | **43.18 (5.14)** | **39.14 (4.68)** | **<0.001** | **41.73 (5.12)** | **40.95 (5.16)** | **0.003** | 40.98 (5.43) | 41.44 (4.43) | 0.158 |
| **TFA, % energy** | **36.17 (5.58)** | **38.46 (4.65)** | **<0.001** | **37.93 (5.74)** | **37.07 (4.70)** | **0.003** | **39.53 (5.12)** | **34.87 (4.75)** | **<0.001** | **38.13 (5.14)** | **36.52 (5.35)** | **<0.001** | 37.50 (5.60) | 37.05 (4.44) | 0.068 |
| **SFA, % energy** | **10.46 (1.86)** | **10.98 (1.77)** | **<0.001** | 10.78 (1.99) | 10.87 (1.65) | 0.335 | **11.31 (1.90)** | **10.26 (1.72)** | **<0.001** | **11.48 (1.71)** | **10.21 (1.82)** | **<0.001** | **9.71 (1.66)** | **11.76 (1.55)** | **<0.001** |
| **Cholesterol, % energy** | **0.35 (0.14)** | **0.33 (0.10)** | **0.001** | **0.33 (0.14)** | **0.37 (0.12)** | **<0.001** | **0.34 (0.13)** | **0.37 (0.14)** | **<0.001** | **0.41 (0.12)** | **0.29 (0.13)** | **<0.001** | **0.31 (0.13)** | **0.39 (0.12)** | **<0.001** |
| **Carbohydrates, % energy** | **45.07 (7.17)** | **41.05 (6.19)** | **<0.001** | 42.99 (7.57) | 42.43 (6.22) | 0.141 | **40.15 (6.68)** | **46.19 (6.48)** | **<0.001** | **41.56 (6.74)** | **44.30 (7.11)** | **<0.001** | **44.74 (7.19)** | **41.48 (5.88)** | **<0.001** |
| **Dietary fiber, g/1000 kcal** | **6.51 (1.69)** | **7.29 (1.49)** | **<0.001** | **6.95 (1.76)** | **6.60 (1.53)** | **<0.001** | **6.41 (1.79)** | **7.04 (1.58)** | **<0.001** | **6.36 (1.64)** | **6.92 (1.69)** | **<0.001** | **7.80 (1.42)** | **5.71 (1.45)** | **<0.001** |
|  |  |  |  |  |  |  |  |  |  |  |  |  |  |  |  |
| **Third trimester** |  |  |  |  |  |  |  |  |  |  |  |  |  |  |  |
| **TEI (100kcal/d)** | **21.20 (6.04)** | **22.02 (4.74)** | **0.006** | 21.78 (5.88) | 21.78 (4.66) | 0.962 | 21.39 (5.54) | 21.10 (5.26) | 0.677 | 20.78 (4.91) | 21.14 (5.91) | 0.611 | **21.52 (5.45)** | **20.85 (4.96)** | **0.009** |
| **Protein, % energy** | **16.57 (2.91)** | **18.90 (2.70)** | **<0.001** | **17.42 (3.05)** | **18.61 (2.60)** | **<0.001** | **18.53 (2.86)** | **16.76 (2.89)** | **<0.001** | **18.70 (2.53)** | **16.87 (3.16)** | **<0.001** | **16.51 (3.00)** | **19.05 (2.37)** | **<0.001** |
| **Total fat, % energy** | **40.33 (5.68)** | **41.45 (4.51)** | **<0.001** | 41.12 (5.54) | 41.22 (4.49) | 0.719 | **41.95 (5.34)** | **39.03 (4.83)** | **<0.001** | **41.29 (4.88)** | **40.65 (5.45)** | **0.013** | **40.51 (5.40)** | **41.40 (4.35)** | **0.003** |
| **TFA, % energy** | **36.06 (5.66)** | **37.89 (4.63)** | **<0.001** | 37.41 (5.55) | 37.18 (4.63) | 0.419 | **38.30 (5.28)** | **34.76 (4.83)** | **<0.001** | **37.74 (4.80)** | **36.17 (5.55)** | **<0.001** | 37.07 (5.56) | 37.04 (4.30) | 0.788 |
| **SFA, % energy** | **10.63 (2.02)** | **10.84 (1.85)** | **0.021** | **10.81 (2.07)** | **11.03 (1.70)** | **0.035** | **11.16 (2.00)** | **10.32 (1.91)** | **<0.001** | **11.55 (1.71)** | **10.33 (2.04)** | **<0.001** | **9.65 (1.67)** | **11.97 (1.52)** | **<0.001** |
| **Cholesterol, % energy** | **0.35 (0.13)** | **0.33 (0.10)** | **0.011** | **0.33 (0.12)** | **0.37 (0.11)** | **<0.001** | 0.35 (0.11) | 0.36 (0.13) | 0.075 | **0.40 (0.11)** | **0.31 (0.12)** | **<0.001** | **0.32 (0.11)** | **0.39 (0.11)** | **<0.001** |
| **Carbohydrates, % energy** | **44.70 (7.51)** | **41.46 (6.39)** | **<0.001** | **43.18 (7.46)** | **41.81 (6.18)** | **<0.001** | **41.12 (7.01)** | **45.94 (6.66)** | **<0.001** | **41.59 (6.46)** | **44.21 (7.47)** | **<0.001** | **44.79 (7.33)** | **41.08 (5.75)** | **<0.001** |
| **Dietary fiber, g/1000 kcal** | **6.25 (1.77)** | **7.34 (1.50)** | **<0.001** | **6.88 (1.80)** | **6.51 (1.60)** | **<0.001** | **6.50 (1.81)** | **6.88 (1.68)** | **<0.001** | **6.23 (1.74)** | **6.80 (1.77)** | **<0.001** | **7.91 (1.37)** | **5.50 (1.45)** | **<0.001** |
| **P*-trends were assessed by modeling the median value of the tertiles in the linear regression analysis. Abbreviations: TEI, total energy intake; TFA, total fatty acids; SFA, saturated fatty acids; T, tertile. | | | | | | | | | | | | | | | |

| **Supplementary Table 5. Adjusted associations of maternal dietary patterns in the second and third trimester of pregnancy with offspring birth weight.** | | |
| --- | --- | --- |
| **Dietary patterns** | **Birth weight** | |
|  | **Beta (95% CI)** | **FDR-*p*** |
| **Second trimester** |  |  |
| **Vegetables and fish** | -8.93 (-22.61, 4.74) | 0.468 |
| **Animal internal organs, thallophyte and shellfish** | 0.33 (-9.00, 9.67) | 0.957 |
| **Fruits and refined grains** | 2.80 (-10.50, 16.10) | 0.953 |
| **Snack and less eggs** | -8.36 (-20.69, 3.98) | 0.455 |
| **Meat and less nuts** | 8.41 (-3.72, 20.53) | 0.455 |
|  |  |  |
| **Third trimester** |  |  |
| **Vegetables and fish** | -4.06 (-17.61, 9.49) | 0.867 |
| **Animal internal organs, thallophyte and shellfish** | 2.15 (-7.99, 12.29) | 0.953 |
| **Fruits and refined grains** | 6.68 (-5.32, 18.68) | 0.551 |
| **Snack and less eggs** | -8.39 (-21.69, 4.92) | 0.479 |
| **Meat and less nuts** | 6.93 (-5.20, 19.07) | 0.551 |
| Analyses were adjusted for mode of conception, area of residence, household income, maternal education, maternal age at conception, maternal pre-pregnancy BMI, parity, chronic diabetes, GDM, TEI, GW at delivery and infant sex. In addition, the dietary patterns were adjusted for each other. Abbreviations: HC, head circumference; AC, abdominal circumference; FL, femur length; EFW, estimated fetal weight; FDR, false discovery rate; TEI, total energy intake; GDM, gestational diabetes mellitus; GW, gestational week. | | |

| **Supplementary Table 6. Adjusted associations of maternal dietary patterns in the second and third trimester of pregnancy with fetal growth indicators in women without chronic diabetes and GDM.** | | | | | | | | |  |
| --- | --- | --- | --- | --- | --- | --- | --- | --- | --- |
| **Dietary patterns** | **HC Z-score** | | **AC Z-score** | | **FL Z-score** | | **FL Z-score** | | |
|  | **Beta (95% CI)** | **FDR-*p*** | **Beta (95% CI)** | **FDR-*p*** | **Beta (95% CI)** | **FDR-*p*** | **Beta (95% CI)** | **FDR-*p*** | |
| **Second trimester** |  |  |  |  |  |  |  |  | |
| **Vegetables and fish** | **-0.09 (-0.12, -0.05)** | **<0.001** | 0.01 (-0.02, 0.04) | 0.752 | -0.01 (-0.04, 0.02) | 0.745 | -0.02 (-0.05, 0.02) | 0.622 | |
| **Animal internal organs, thallophyte and shellfish** | 0.03 (0.01, 0.06) | 0.102 | 0.01 (-0.02, 0.03) | 0.693 | 0.001 (-0.02, 0.02) | 0.996 | 0.01 (-0.01, 0.04) | 0.613 | |
| **Fruits and refined grains** | -0.01 (-0.04, 0.03) | 0.871 | 0.02 (-0.01, 0.05) | 0.613 | -0.01 (-0.04, 0.02) | 0.746 | 0.01 (-0.02, 0.05) | 0.622 | |
| **Snack and less eggs** | **-0.06 (-0.10, -0.03)** | **0.002** | -0.01 (-0.04, 0.02) | 0.622 | -0.01 (-0.04, 0.02) | 0.643 | -0.03 (-0.06, 0.01) | 0.252 | |
| **Meat and less nuts** | 0.04 (0.01, 0.08) | 0.087 | -0.01 (-0.04, 0.02) | 0.757 | 0.01 (-0.03, 0.04) | 0.849 | 0.004 (-0.03, 0.04) | 0.892 | |
|  |  |  |  |  |  |  |  |  | |
| **Third trimester** |  |  |  |  |  |  |  |  | |
| **Vegetables and fish** | **-0.06 (-0.10, -0.02)** | **0.013** | 0.004 (-0.03, 0.04) | 0.988 | 0.001 (-0.04, 0.04) | 0.988 | -0.01 (-0.04, 0.02) | 0.958 | |
| **Animal internal organs, thallophyte and shellfish** | 0.02 (-0.01, 0.05) | 0.739 | -0.004 (-0.03, 0.02) | 0.988 | 0.01 (-0.02, 0.03) | 0.988 | 0.002 (-0.02, 0.03) | 0.988 | |
| **Fruits and refined grains** | -0.02 (-0.06, 0.02) | 0.842 | 0.004 (-0.03, 0.04) | 0.988 | 0.003 (-0.03, 0.04) | 0.988 | -0.002 (-0.04, 0.03) | 0.988 | |
| **Snack and less eggs** | -0.05 (-0.08, -0.01) | 0.091 | -0.01 (-0.04, 0.03) | 0.988 | -0.02 (-0.05, 0.02) | 0.881 | -0.02 (-0.05, 0.02) | 0.881 | |
| **Meat and less nuts** | 0.04 (0.01, 0.08) | 0.059 | 0.001 (-0.03, 0.03) | 0.988 | 0.01 (-0.02, 0.05) | 0.901 | 0.01 (-0.02, 0.04) | 0.941 | |
| Analyses were adjusted for mode of conception, area of residence, household income, maternal education, maternal age at conception, maternal pre-pregnancy BMI, parity, TEI, and infant sex. In addition, the dietary patterns were adjusted for each other. Abbreviations: HC, head circumference; AC, abdominal circumference; FL, femur length; EFW, estimated fetal weight; FDR, false discovery rate; TEI, total energy intake; GDM, gestational diabetes mellitus. | | | | | | | | | |

| **Supplementary Table 7. Adjusted associations of maternal dietary patterns in the second and third trimester of pregnancy with fetal growth indicators in women without anemia during pregnancy.** | | | | | | | | | | | |
| --- | --- | --- | --- | --- | --- | --- | --- | --- | --- | --- | --- |
| **Dietary patterns** | **HC Z-score** | | | **AC Z-score** | | | **FL Z-score** | | | **EFW Z-score** | |
|  | **Beta (95% CI)** | **FDR-*p*** | **Beta (95% CI)** | | **FDR-*p*** | **Beta (95% CI)** | | **FDR-*p*** | **Beta (95% CI)** | | **FDR-*p*** |
| **Second trimester** |  |  |  | |  |  | |  |  | |  |
| **Vegetables and fish** | **-0.09 (-0.12, -0.06)** | **<0.001** | 0.01 (-0.02, 0.04) | | 0.797 | 0.003 (-0.03, 0.03) | | 0.905 | -0.02 (-0.05, 0.01) | | 0.464 |
| **Animal internal organs, thallophyte and shellfish** | **0.04 (0.02, 0.06)** | **0.006** | 0.01 (-0.01, 0.03) | | 0.457 | -0.004 (-0.03, 0.02) | | 0.833 | 0.02 (-0.01, 0.04) | | 0.153 |
| **Fruits and refined grains** | -0.01 (-0.04, 0.02) | 0.719 | 0.01 (-0.02, 0.04) | | 0.695 | -0.01 (-0.04, 0.02) | | 0.797 | 0.01 (-0.02, 0.03) | | 0.816 |
| **Snack and less eggs** | **-0.05 (-0.08, -0.02)** | **0.004** | -0.01 (-0.04, 0.02) | | 0.719 | -0.01 (-0.04, 0.02) | | 0.797 | -0.02 (-0.05, 0.01) | | 0.276 |
| **Meat and less nuts** | 0.03 (-0.01, 0.06) | 0.250 | -0.02 (-0.05, 0.01) | | 0.362 | 0.01 (-0.02, 0.04) | | 0.653 | -0.01 (-0.04, 0.02) | | 0.719 |
|  |  |  |  | |  |  | |  |  | |  |
| **Third trimester** |  |  |  | |  |  | |  |  | |  |
| **Vegetables and fish** | **-0.06 (-0.09, -0.02)** | **0.008** | 0.02 (-0.01, 0.05) | | 0.612 | 0.01 (-0.03, 0.04) | | 0.986 | 0.002 (-0.03, 0.03) | | 0.986 |
| **Animal internal organs, thallophyte and shellfish** | 0.01 (-0.02, 0.04) | 0.741 | -0.01 (-0.03, 0.01) | | 0.701 | -0.02 (-0.04, 0.01) | | 0.612 | -0.01 (-0.03, 0.02) | | 0.877 |
| **Fruits and refined grains** | -0.03 (-0.06, -0.01) | 0.158 | 0.01 (-0.02, 0.03) | | 0.938 | -0.002 (-0.03, 0.03) | | 0.986 | -0.003 (-0.03, 0.02) | | 0.986 |
| **Snack and less eggs** | -0.04 (-0.07, -0.01) | 0.163 | 0.01 (-0.02, 0.04) | | 0.741 | -0.02 (-0.05, 0.02) | | 0.660 | 0.001 (-0.03, 0.03) | | 0.986 |
| **Meat and less nuts** | **0.04 (0.01, 0.07)** | **0.023** | -0.002 (-0.03, 0.02) | | 0.986 | 0.03 (-0.01, 0.05) | | 0.255 | 0.01 (-0.02, 0.04) | | 0.769 |
| Analyses were adjusted for mode of conception, area of residence, household income, maternal education, maternal age at conception, maternal pre-pregnancy BMI, parity, chronic diabetes, GDM, TEI, and infant sex. In addition, the dietary patterns were adjusted for each other. Abbreviations: HC, head circumference; AC, abdominal circumference; FL, femur length; EFW, estimated fetal weight; FDR, false discovery rate; TEI, total energy intake; GDM, gestational diabetes mellitus. | | | | | | | | | | | |

| **Supplementary Table 8. Stratified analyses of associations between maternal dietary patterns in the second and third trimester of pregnancy and fetal growth indicators.** | | | | | | | | | | | | |
| --- | --- | --- | --- | --- | --- | --- | --- | --- | --- | --- | --- | --- |
| **Dietary patterns** | **Groups** | | **HC_zscore** | | **AC_zscore** | | **FL_zscore** | | | | **EFW_zscore** | |
|  |  |  | **Beta (95% CI)** | ***p**** | **Beta (95% CI)** | ***p**** | | **Beta (95% CI)** | ***p**** | **Beta (95% CI)** | | ***p**** |
| **Second trimester** |  |  | |  |  |  | |  |  |  | |  |
| **Vegetables and fish** | ART | **-0.12 (-0.16, -0.07)** | | 0.429 | 0.004 (-0.04, 0.04) | 0.801 | | -0.01 (-0.05, 0.04) | 0.731 | -0.03 (-0.07, 0.01) | | 0.770 |
|  | SP | **-0.06 (-0.11, -0.02)** | |  | -0.003 (-0.04, 0.03) |  | | 0.002 (-0.04, 0.04) |  | -0.02 (-0.06, 0.02) | |  |
| **Animal internal organs, thallophyte and shellfish** | ART | 0.04 (0.001, 0.07) | | 0.855 | 0.01 (-0.02, 0.03) | 0.801 | | -0.01 (-0.04, 0.02) | 0.687 | 0.01 (-0.02, 0.04) | | 0.567 |
|  | SP | **0.05 (0.02, 0.07)** | |  | 0.02 (-0.01, 0.04) |  | | 0.01 (-0.02, 0.04) |  | 0.03 (0.001, 0.06) | |  |
| **Fruits and refined grains** | ART | -0.01 (-0.05, 0.03) | | 0.855 | 0.04 (-0.001, 0.07) | 0.309 | | 0.01 (-0.03, 0.05) | 0.687 | 0.03 (-0.01, 0.07) | | 0.567 |
|  | SP | -0.01 (-0.05, 0.03) | |  | -0.004 (-0.04, 0.03) |  | | -0.01 (-0.05, 0.03) |  | -0.005 (-0.04, 0.03) | |  |
| **Snack and less eggs** | ART | -0.03 (-0.08, 0.01) | | 0.669 | 0.03 (-0.01, 0.07) | 0.113 | | 0.01 (-0.03, 0.05) | 0.687 | 0.02 (-0.02, 0.05) | | 0.149 |
|  | SP | **-0.06 (-0.10, -0.02)** | |  | -0.03 (-0.06, 0.01) |  | | -0.02 (-0.06, 0.01) |  | -0.04 (-0.08, -0.01) | |  |
| **Meat and less nuts** | ART | 0.05 (0.01, 0.09) | | 0.669 | -0.01 (-0.04, 0.03) | 0.801 | | 0.02 (-0.02, 0.06) | 0.687 | 0.01 (-0.03, 0.05) | | 0.567 |
|  | SP | 0.02 (-0.02, 0.06) | |  | -0.02 (-0.05, 0.02) |  | | 0.01 (-0.03, 0.04) |  | -0.01 (-0.05, 0.03) | |  |
|  |  |  | |  |  |  | |  |  |  | |  |
| **Third trimester** |  |  | |  |  |  | |  |  |  | |  |
| **Vegetables and fish** | ART | **-0.09 (-0.14, -0.04)** | | 0.199 | 0.01 (-0.03, 0.05) | 0.959 | | 0.003 (-0.04, 0.05) | 0.955 | -0.01 (-0.06, 0.03) | | 0.885 |
|  | SP | -0.02 (-0.06, 0.03) | |  | 0.01 (-0.03, 0.05) |  | | 0.01 (-0.04, 0.05) |  | 0.004 (-0.03, 0.04) | |  |
| **Animal internal organs, thallophyte and shellfish** | ART | 0.03 (-0.01, 0.06) | | 0.524 | -0.01 (-0.05, 0.02) | 0.959 | | -0.01 (-0.04, 0.03) | 0.955 | -0.005 (-0.04, 0.03) | | 0.994 |
|  | SP | 0.01 (-0.02, 0.04) | |  | -0.01 (-0.04, 0.02) |  | | -0.01 (-0.04, 0.02) |  | -0.005 (-0.03, 0.02) | |  |
| **Fruits and refined grains** | ART | -0.04 (-0.08, 0.01) | | 0.523 | -0.01 (-0.04, 0.03) | 0.959 | | 0.002 (-0.04, 0.04) | 0.955 | -0.01 (-0.05, 0.02) | | 0.846 |
|  | SP | -0.01 (-0.04, 0.03) | |  | 0.01 (-0.02, 0.05) |  | | 0.004 (-0.03, 0.04) |  | 0.01 (-0.02, 0.04) | |  |
| **Snack and less eggs** | ART | -0.01 (-0.06, 0.04) | | 0.523 | 0.02 (-0.02, 0.06) | 0.959 | | -0.01 (-0.06, 0.04) | 0.955 | 0.01 (-0.03, 0.06) | | 0.846 |
|  | SP | -0.05 (-0.09, -0.01) | |  | -0.01 (-0.04, 0.03) |  | | -0.04 (-0.08, -0.001) |  | -0.02 (-0.05, 0.02) | |  |
| **Meat and less nuts** | ART | 0.04 (-0.01, 0.08) | | 0.705 | 0.001 (-0.04, 0.04) | 0.959 | | 0.02 (-0.02, 0.06) | 0.955 | 0.01 (-0.03, 0.05) | | 0.994 |
|  | SP | 0.05 (0.01, 0.09) | |  | 0.004 (-0.03, 0.04) |  | | 0.04 (-0.01, 0.07) |  | 0.02 (-0.02, 0.05) | |  |
| Analyses were adjusted for area of residence, household income, maternal education, maternal age at conception, maternal pre-pregnancy BMI, parity, chronic diabetes, GDM, TEI, and infant sex. In addition, the dietary patterns were adjusted for each other. * *p* value for heterogeneity test. Abbreviations: ARTP, assisted reproductive technology pregnancy; SP, spontaneous pregnancy; HC, head circumference; AC, abdominal circumference; FL, femur length; EFW, estimated fetal weight; FDR, false discovery rate; TEI, total energy intake; GDM, gestational diabetes mellitus. | | | | | | | | | | | | |

| **Supplementary Table 9. Adjusted associations of food groups in the second and third trimester with fetal HC in different GWs.** | | | |
| --- | --- | --- | --- |
| **Trimester** | **Food groups** | **HC Z-score** | |
|  |  | **Beta ( 95% CI )** | **FDR*-p*** |
| **Second** | **Refined cereals** | 0.06 (-0.01, 0.13) | 0.378 |
|  | **Tubers and whole grains** | 0.01 (-0.03, 0.05) | 0.968 |
|  | **Fried food** | 0.01 (-0.03, 0.05) | 0.883 |
|  | **Sweets** | -0.02 (-0.06, 0.01) | 0.520 |
|  | **Beans** | 0.04 (0.01, 0.07) | 0.125 |
|  | **Vegetables** | -0.06 (-0.14, 0.01) | 0.378 |
|  | **Thallophyte** | -0.02 (-0.06, 0.02) | 0.631 |
|  | **Citrus** | 0.01 (-0.01, 0.03) | 0.566 |
|  | **Pome, berry and melon** | -0.03 (-0.08, 0.02) | 0.586 |
|  | **Nut** | -0.03 (-0.06, -0.01) | 0.255 |
|  | **Eggs** | **0.06 (0.02, 0.10)** | **0.043** |
|  | **Dairy** | 0.04 (0.01, 0.06) | 0.094 |
|  | **Meat** | 0.01 (-0.03, 0.06) | 0.816 |
|  | **Animal internal organs** | 0.02 (-0.01, 0.05) | 0.404 |
|  | **Fish and shrimp** | **-0.07 (-0.11, -0.03)** | **0.012** |
|  | **Shellfish** | 0.04 (-0.01, 0.08) | 0.378 |
|  |  |  |  |
| **Third** | **Refined cereals** | 0.04 (-0.02, 0.10) | 0.672 |
|  | **Tubers and whole grains** | -0.02 (-0.07, 0.03) | 0.791 |
|  | **Fried food** | -0.002 (-0.04, 0.04) | 0.970 |
|  | **Sweets** | -0.03 (-0.07, 0.01) | 0.591 |
|  | **Beans** | 0.01 (-0.02, 0.05) | 0.791 |
|  | **Vegetables** | -0.10 (-0.18, -0.01) | 0.174 |
|  | **Thallophyte** | 0.02 (-0.03, 0.07) | 0.791 |
|  | **Citrus** | -0.01 (-0.03, 0.01) | 0.747 |
|  | **Pome, berry and melon** | -0.05 (-0.10, -0.01) | 0.214 |
|  | **Nut** | **-0.04 (-0.07, -0.01)** | **0.036** |
|  | **Eggs** | 0.04 (-0.02, 0.09) | 0.672 |
|  | **Dairy** | 0.01 (-0.03, 0.04) | 0.942 |
|  | **Meat** | 0.01 (-0.04, 0.06) | 0.894 |
|  | **Animal internal organs** | 0.001 (-0.03, 0.03) | 0.970 |
|  | **Fish and shrimp** | -0.03 (-0.07, 0.02) | 0.730 |
|  | **Shellfish** | 0.002 (-0.05, 0.05) | 0.970 |
| Analyses were adjusted for mode of conception, area of residence, household income, maternal education, maternal age at conception, maternal pre-pregnancy BMI, parity, chronic diabetes, GDM, TEI, and infant sex. In addition, the food groups were adjusted for each other. Abbreviations: HC, head circumference; GW, gestational week; FDR, false discovery rate; TEI, total energy intake; GDM, gestational diabetes mellitus. | | | |
